# Experimental myocarditis in rat can be detected and monitored by cardiac magnetic resonance imaging performed on a clinical 3.0 T scanner

**DOI:** 10.1186/1532-429X-13-S1-O49

**Published:** 2011-02-02

**Authors:** Shunit Rinkevich-Shop, Natalie Landa-Rouben, Fred Epstein, Tamar Ben-Mordechai, Arnon Afek, Micha S Feinberg, Orly Goitein, Eli Konen, Tammar Kushnir, Jonathan Leor

**Affiliations:** 1 Neufeld Cardiac Research Institute, Tel Aviv University, Tel- Hashomer, Israel; 2 Departments of Radiology and Biomedical Engineering, University of Virginia, Virginia, VA, USA; 3 Sheba Medical Center, Tel- Hashomer, Israel; 4 Diagnostic Imaging Department, Sheba Medical Center, Tel- Hashomer, Israel

## Background

We aimed to compare cardiac magnetic resonance (CMR) imaging, using a clinical whole body 3T system, with histopathological measurement and 2D echocardiography as a method for the quantitative evaluation of the extent of myocardial involvement and function in a rat model of autoimmune myocarditis.

## Methods and results

**Figure 1:**
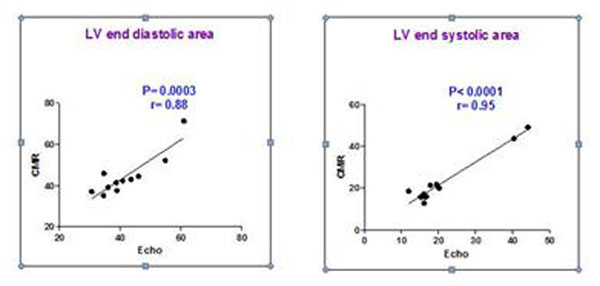
Correlation between echo and CMR in the assessment of LV end diastolic and systolic area in rats with myocarditis.

Male Lewis rats (n=11) were subjected to myosin immunization and developed autoimmune myocarditis. Approximately 3 weeks later, rats with myocarditis underwent CMR scan and subsequently, histopathological evaluation. Rats with myocarditis showed pericardial thickening, effusion, and LV wall motion abnormalities with septal hypokinesis. Short axis views showed patchy delayed enhancement of epicardial segments, with distribution mainly located within the inferolateral LV wall including the septum. This increased signal/hyperenhancement defines focal areas of myocardial fibrosis/edema and/or necrosis, highly suggestive of inflammation. Additionally, the presence of large pericardial effusion provides supportive evidence for the existence of peri- myocarditis. Indeed, high correlation was found between CMR examination results and histological findings. Furthermore, high correlation was found between CMR examination and echocardiography (Figure 1)

## Conclusions

Experimental myocarditis in rat can be detected and monitored by CMR performed on a clinical 3.0 T scanner. The overall advantages of CMR, mostly its high measurement accuracy and reproducibility, make it an ideal technique for monitoring experimental myocarditis and pre-clinical evaluation of novel therapies.

